# Evaluating Metrics to Improve Population Health

**Published:** 2010-06-15

**Authors:** Linda T. Bilheimer

**Affiliations:** Centers for Disease Control and Prevention

## Introduction

The 7 metrics articles in this issue of *Preventing Chronic Disease* address the following topics: public health policy (1), health care access and quality (2), social and economic determinants (3), health behaviors (4), environmental metrics (5), population health outcomes (6), and health inequalities (7). The articles differ in the degree to which they establish a conceptual framework for linking metrics to rewards to improve population health. Their different perspectives raise questions of whether these metrics should meet certain criteria, regardless of domain, or whether some flexibility in the criteria for assessing metrics is necessary and desirable. Questions that arise in establishing such criteria relate to structure and function as well as data availability.

## Structure and Function of Population Health Metrics

In establishing a framework for linking performance incentives to population health metrics, researchers must answer multiple questions.

**Are the measures actionable?** If so, at what level and by whom? Although these articles focus on community-level interventions, not all the suggested metrics seem to be actionable at that level. Nor would they necessarily be applicable for the range of organizations and agencies that affect population health in communities. A related question is whether all metrics should be actionable. Some of the suggested metrics — such as those in the socioeconomic domain — are contextual variables that influence health status and health care access and use and should be taken into account in assessing community-level performance. Such metrics may be actionable at the state or national levels, rather than the community level.

**Are the measures sensitive to interventions?** If so, within what time frame? A system for rewarding initiatives to improve population health needs metrics that not only respond to interventions but also do so in a realistic time frame for incentives to be meaningful. As the population health outcomes article points out, for example, life expectancy and age-adjusted mortality are measures of population health that are amenable to intervention, but not necessarily in a realistic time frame (6). Also important is whether metrics are sensitive to interventions at different levels: upstream, midstream, and downstream. Those terms may have different meanings in different contexts and domains. The authors of the public health policy article (1), for example, describe upstream approaches as those with the potential to affect large populations through regulation, increased access, or economic incentives. They classify interventions in organizations, such as worksite health improvement programs, as midstream, and individual-level behavioral approaches as downstream. The environmental metrics article (5) contrasts environmental factors, such as air quality, that affect human health directly and proximately with upstream factors, such as a community's energy sources, that affect health indirectly. In the social and economic determinants article (3), upstream refers to the social determinants of health.

**Are the measures affected by population migration?** This question is of particular relevance for analyzing community-level health metrics, especially longer-term, because the composition of local populations can change substantially. Changes in life expectancy over time at the community level, for example, will reflect changes in population composition as well as changes in underlying health status.

**Are the measures easily understood by collaborating organizations, policy makers, and the public?** The need for simplicity and easy comprehension is a common theme in several of the articles (1,5-7). When complex measures — such as the univariate inequalities measure, which assesses overall inequality across a population, regardless of association with other attributes (7) — are proposed, one question that arises is whether an effective communications strategy could facilitate understanding. Although metrics linking workforce health status and productivity have been established, the business case for addressing the health of communities may be less clear (8).

**Is the meaning of an increase or decrease in a measure unambiguous?** For most of the suggested measures in the articles, a change in a given direction can be readily interpreted as positive or negative. For some measures, however, the implications of a change in a particular direction may be unclear. In the case of participation in social welfare programs, for example, higher participation rates may reflect increased economic hardship in a community (negative), more effective outreach to the low-income population or more generous eligibility criteria (positive), or both.

**Do the measures stand alone or are they aggregated into an index or summary measure?** The articles differ in the extent to which they recommend aggregation. The outcomes and inequalities articles (6,7) promote the use of summary measures — exclusively in the case of inequalities — and the socioeconomic determinants article (3) suggests the possibility of using an index or identifying complex measures by using factor or principal component analyses. A major advantage of a summary measure is parsimony; having a large number of metrics can lead to loss of focus, which a single measure avoids. In the case of a weighted measure, however, reaching agreement on the appropriate weights may be difficult and ultimately subjective. Several of the previous questions, moreover, have particular bearing on these more complex types of measures. Is their meaning clear to users? Are they readily actionable? Are they responsive to interventions? Does a change in a given direction have an unambiguous interpretation? The answers to those questions depend in part on whether a complex measure can be disaggregated into meaningful components. In that regard, the inequalities article (7) provides an example of how to isolate the contributions of different attributes to an overall measure of inequality, thereby guiding intervention priorities.

**Are the measures uniform across communities?** Although measures need to be comparable across communities, some flexibility may be necessary. In the case of health determinants, the particular domain is pertinent. One could make a case for standard measures of behavioral risks, for example, because such risks are not community-specific. However, environmental issues vary widely among communities, leading those authors to suggest that communities should be involved in both defining and using environmental metrics (5). A possible approach, at least for some domains, is to have a core set of standard measures, with additional measures selected by the community.

**To what extent do measures address disparities as well as overall burden?** The articles adopt different perspectives toward disparities. The health care article (2) proposes a single measure to track disparities, whereas others (1,5,6) suggest that the ability to identify and monitor disparities should be an integral feature of all measures. However, the health policy article (1) points out how disparities assessment is limited in that domain. Notably, most of the articles assume a bivariate approach to disparities measurement rather than the univariate approach that the inequalities article (7) recommends.

**Can unintended consequences be tracked?** None of the articles mentions the potential for unintended consequences that may result from the use of certain metrics in an accountability-based system — an issue that has arisen in the clinical setting. If incentives reward improvements in specific population health measures, tracking additional metrics may be necessary to ensure that any improvements do not come at the cost of deterioration in other population health domains.

## Data Availability for Population Health Metrics

Having reliable and valid measures to provide incentives to improve population health depends on the availability of high-quality, timely data. A consideration is whether data availability should drive the choice of metrics or whether alternative data strategies should be explored. The articles have different perspectives on this issue, reflecting the variation in data availability in domains, which in turn reflects such factors as changing survey technologies (including the shift to multimode surveys), the rapid development of health information technology, the extent of administrative data systems, data linkage and integration, and the potential for modeling. Several questions have bearing on data decisions and choice of metrics.

**Do the available data correspond to the geographic level of the intervention?** This question is particularly relevant to community-level interventions because many national surveys do not have sufficient sample sizes to produce local estimates. As the health behaviors article points out (4), even if local estimates can be produced, the standard errors may be so large that they make responses to interventions difficult to detect. For the same reason, cross-sectional differences among communities may also be difficult to identify, and community rankings based only on point estimates may be quite misleading. The heavy microdata demands of the univariate approach to disparities measurement that the inequalities article promotes (7) would make that approach particularly difficult to implement at the local level.

**How timely are the data?** Rewarding performance requires recent data that are released on a regular basis. The need for current data may affect strategies for addressing small sample sizes in communities; aggregating data over several years to boost sample size limits the sensitivity of a measure to detect changes in response to an intervention.

**Are the measures reliable and valid?** Although the articles mention the need for reliability and validity, they do not indicate how they would assess the measures that they propose.

**Can the measures be produced for population subgroups?** Tracking racial/ethnic, socioeconomic, and other disparities requires far more extensive data and much larger survey sample sizes than does monitoring population health overall. These data demands pose substantial challenges for identifying and tracking disparities at the community level.

**Are indirect methods of estimation appropriate?** New tools for indirect estimation, including data integration and linkage, Bayesian estimation, and systems modeling, offer potential strategies for developing community-level estimates, including estimates for subpopulations. The environment article (5) provides an example, highlighting the role of geographic information systems in linking health determinants and outcomes over spatial scales. Concerns include how to assess the reliability and validity of indirect estimates and how to communicate findings effectively. Skepticism about modeled estimates may limit their use for policy decisions.

**Should data reporting be part of an incentive-based population health improvement system? **This idea was raised in a recent Institute of Medicine report on addressing disparities in health care quality (9) and deserves discussion in a population health context.

## Conclusion

As policy makers consider strategies to promote improvements in population health, measurement may provide powerful incentives for change, but selecting reliable and valid health metrics that can be tracked consistently across communities is challenging. The 7 articles in this issue illustrate many of the complexities that policy makers must consider in selecting such metrics, and the articles lay the groundwork for ongoing discussions on this topic.
